# Diagnostic and therapeutic challenges of EBV-positive mucocutaneous ulcer: a case report and systematic review of the literature

**DOI:** 10.1186/s40164-016-0042-5

**Published:** 2016-04-27

**Authors:** Toni K. Roberts, Xueyan Chen, Jay Justin Liao

**Affiliations:** 1 Department of Hematology Oncology, Fred Hutchinson Cancer Research Center, University of Washington Allied Hospitals, 1100 Fairview Ave N-D5-100, Seattle, WA 98109-1024 USA; 2 Department of Laboratory Medicine, UW Hematopathology Laboratory, University of Washington, Box 358081, 825 Eastlake Ave E, Seattle, WA 98109 USA; 3 Department of Radiation Oncology, University of Washington, 1959 NE Pacific St, 1st floor, NN106, Seattle, WA 98195 USA

**Keywords:** Epstein–Barr virus-positive mucocutaeneous ulcer disease, Epstein–Barr virus, Lymphoproliferative disorder, B-cell neoplasm, Radiation therapy, Rituximab, Immunosenescence

## Abstract

**Background:**

Epstein–Barr virus-positive mucocutaneous ulcer (EBVMCU) is a recently recognized B cell lymphoproliferative disorder that is driven by latent EBV infection and causes discrete ulcerations in the oropharynx, gastrointestinal tract, and skin. Local attenuation of immunosurveillance associated with iatrogenic immunosuppressant use, primary immunodeficiency, or age-associated immunosenescence has been implicated as a predisposing factor. This disorder is likely under reported, as it was only first defined in 2010 and shares histological features with other B-cell proliferative neoplasms. The first case series that described EBVMCU suggested that EBVMCU is generally self-limited and is likely to resolve without treatment. Since that publication, additional cases have been reported that describe a more heterogeneous clinical course, often requiring aggressive therapy. We now systematically review all published cases of EBVMCU and detail a case of aggressive and progressive EBVMCU, including diagnostic and management challenges, as well as successful treatment with radiation therapy.

**Case presentation:**

A forty-nine year old woman presented with painful and debilitating multifocal oral EBVMCU that initially responded to four weekly doses of rituximab. Her disease relapsed within 3 months and continued to progress and cause significant morbidity. She was successfully treated with local external beam radiation therapy of 30 Gy in 15 fractions, with duration of response of at least 6 months.

**Conclusions:**

We suggest that although many patients with EBVMCU experience a self-limited course, for others EBVMCU can be a debilitating, persistent disorder that requires aggressive therapy to prevent disease progression. CD20- and CD30-directed antibody therapy, local radiation therapy, local surgical excision, systemic chemotherapy, and a combination of these therapies have all been successfully used to treat EBVMCU with high rates of durable clinical remission. As EBVMCU is not currently included in the 2008 WHO classification of lymphoproliferative disorders and no evidence-based guidelines or expert opinions have been proposed to guide therapy, this case report and systematic review provides a foundation on which to guide therapeutic decisions.

## Background

### EBV biology

Epstein–Barr virus (EBV), also known as human herpes virus 4, is one of eight viruses within the human herpes virus family that infects humans and is directly transferred between humans through saliva [1]. EBV preferentially infects B-cells through binding of viral envelope glycoprotein gp350/220 to CD21 (complement receptor type 2) on B-cells [2]. This binding facilitates the interaction of viral glycoprotein gp42 with B-cell major histocompatibility complex (MHC) class II, thereby triggering fusion of the viral envelope with the B-cell plasma membrane and allowing EBV deoxyribonucleic acid (DNA) entry into the B-cell [3]. Although a primary lytic infection may result, more often viral latency is established when the 172 kilobase linear, double stranded viral DNA circularizes within the cell and persists as an episome in the nucleus of an infected B-cell [4].

During latency, multiple EBV-encoded ribonucleic acids (RNAs) and proteins may be transcribed and expressed that promote lymphocyte proliferation and survival and maintain viral latency [5]. Newly infected naïve B-cells may express all of the EBV latency genes and exist in a highly immunogenic state referred to as latency III [5]. After initial proliferation, some EBV-infected naïve B-cells undergo germinal center reactions that increase the clonal expansion and cellular pool of EBV-infected B-cells [6]. Germinal center B-cells transcribe a more limited set of EBV genes, referred to as latency II [5]. When germinal center B-cells differentiate into long lived, resting memory B-cells, a long-term reservoir of EBV infection is established [7] with minimal transcription of viral genes (latency I) [5]. The absence of MHC presentation of viral antigens facilitates viral latency by promoting escape from immunosurveillance. If a resting memory B-cell is activated, it may differentiate into a plasma cell and induce the EBV lytic life cycle and production of new EBV virions capable of infecting additional B-cells and propagating viral latency indefinitely. B-cell transformation into malignant cells can occur in latently EBV-infected B-cells, with constitutional activation of nuclear factor-κB [8] and apoptosis suppression [9] by viral RNAs and proteins at least partially implicated. Immunosuppression from human immunodeficiency virus (HIV), chemotherapeutics, or immunosuppressive agents as well as age-related immunosenescence may facilitate EBV-associated B-cell transformation by reducing T-cell mediated immunosurveillance and enabling expansion and proliferation of the EBV-infected B-cell reservoir.

### EBV lymphoproliferative disease spectrum

Most individuals are exposed to EBV during the first several decades of life and experience an asymptomatic infection or a self-limited lymphoproliferative infective mononucleosis [10, 11]. Viral latency and long-term persistence are established in the memory B-cell compartment after initial infection [7]. In some individuals EBV may potentiate B-cell transformation and cause a spectrum of EBV-associated lymphoproliferative disorders including Burkitt lymphoma [12], classical Hodgkin lymphoma [13], plasmablastic lymphoma [14], primary effusion lymphoma [15], diffuse large B-cell lymphoma associated with chronic inflammation [16], EBV-positive diffuse large B-cell lymphoma of the elderly [17], lymphomatoid granulomatosis [18], post-transplant lymphoproliferative disorder (PTLD) [19], and EBV-positive mucocutaneous ulcer disease [20]. In addition, T-cells and natural killer (NK) cells can be influenced by surrounding EBV-infected B-cells and can be directly infected with EBV [21, 22] resulting in rare EBV-associated T-cell lymphoproliferative disorders including peripheral T-cell lymphoma [23, 24], angioimmunoblastic T-cell lymphoma [25], extranodal nasal type NK/T-cell lymphoma [26], enteropathy-associated T-cell lymphoma [27, 28], gamma-delta T-cell lymphoma (hepatosplenic and nonhepatosplenic) [29, 30], systemic EBV-positive T-cell lymphoproliferative disease of childhood [31], EBV-associated anaplastic large cell lymphoma [32], and aggressive NK-cell leukemia [33].

### EBV-Positive mucocutaneous ulcer

EBV-positive mucocutaneous ulcer (EBVMCU) was first described and proposed as a distinct clinical entity in 2010 by Dr. Elaine Jaffe’s group at the National Cancer Institute [20]. They described 26 cases in which patients exhibited a common clinical presentation of mucocutaneous ulcers that had a unique histology and immunophenotype among EBV-associated lymphoproliferative disorders (Table 1). EBVMCU was described as shallow, sharply circumscribed mucosal or cutaneous ulcers with an underlying polymorphous infiltration of small lymphocytes, immunoblasts, and atypical larger lymphocytes, and with a variable admixture of scattered plasma cells, eosinophils, and histiocytes. A prominent rim of small T-lymphocytes was also noted at the base of the lesions. The atypical lymphocytes were large and pleomorphic immunoblasts with frequent Hodgkin and Reed-Sternberg (HRS) morphology. Variably sized plasmacytoid apoptotic cells were often seen and angioinvasion and tissue necrosis were variably present. The immunoblasts had a B-cell immunophenotype with uniform expression of CD30, MUM1, PAX5, and OCT-2, and variable CD20, CD45, CD15, CD79a, and BCL-6 expression. EBV was present in the infiltrating small B-cells, plasmacytoid apoptotic cells, and immunoblasts, as demonstrated by EBER-positive in situ hybridization and LMP1 colocalization, suggesting type II latency. Monoclonal immunoglobulin rearrangement was identified in a subset of cases (38.9 %), indicating a clonal EBV-driven B-cell proliferation. Monoclonal or clonally restricted T-cells were also identified in 69 % of the cases, consistent with a restricted T-cell repertoire against EBV-epitopes.

**Table 1:** Pathologic Features of EBVMCU

Histology	Immunophenotype
Shallow, sharply circumscribed ulcers	Immunoblasts often with HRS features: strong positivity for CD10, CD30, MUM1 uniform positivity for PAX5 and OCT-2 variable expression of CD20, CD45, CD15, CD79a, BCL-6, BOB.1
Localized to mucosa of oropharynx and gastrointestinal tract or to skin	Infiltrating lymphocytes: CD4 and CD8 positivity
Polymorphous infiltrate of lymphocytes and immunoblasts	*Clonality*
Variable admixture of scattered plasma cells, eosinophils, and histiocytes	Immunoblasts with HRS features: monoclonal immunoglobulin rearrangement
Medium sized lymphocytes with angulated nuclei	Infiltrating T-lymphocytes: frequent monoclonal or restricted TCR pattern
Pleomorphic immunoblasts with frequent Hodgkin and Reed-Sternberg (HRS) morphology	*Association with EBV*
Plasmacytoid apoptotic cells	Immunoblasts and HRS cells: uniformly EBER1-positive
Variable angioinvasion	Infiltrating T-lymphocytes: frequently EBER1-positive
Variable tissue necrosis	Plasmacytoid apoptotic cells: uniformly EBER1-postive

Since the first description of EBVMCU in 2010, 25 additional cases have been reported (Table 2). In addition, several cases of isolated mucocutaneous ulcers in the setting of immunosuppression were published prior to 2010 and may in fact represent cases of EBVMCU, although thorough pathologic findings were not reported [34–41]. In all 51 published cases, EBV-associated mucocutaneous ulcers were noted to occur in the oropharynx, gastrointestinal tract (GI), or cutaneous skin. The oropharynx was the most common site affected in these cases (41 %), likely because the oropharynx is the most frequent portal of entry for EBV and site of primary infection [1, 42], often within Waldeyer’s ring [43, 44]. Most patients had solitary lesions; however 16 % of patients developed multifocal disease. One of the 51 patients had evidence of active EBV viremia [45], while the remainder of individuals were immunosuppressed either secondary to iatrogenic immunosuppressive agents (methotrexate, azathioprine, cyclosporine, mycophenolate, or tacrolimus) in 56 %, underlying immunodeficiency syndrome (hypogammaglobulinemia, T-cell deficiency) in 4 %, or presumed immunosenescence from advanced age (median age 80 years, range 64–101 years) in 40 %. Interestingly, most of the patients with iatrogenic immunosuppression from immunosuppressive agents were also of advanced age (median 63 years, mode 80 years, range 18–81 years), suggesting that immunosenescence may be a predisposing factor to development of EBVMCU in individuals receiving therapeutic immunosuppression. Both immunosuppression (primary and acquired) and immunosenescence are thought to diminish the EBV-responsive T-cell repertoire and reduce the ability of T-cells to recognize the full range of EBV epitopes [46]. In particular, during immunosenescence, clonal CD8-positive T-cells with a mature memory cell phenotype and reduced functionality accumulate as a result of an age-related diminished capacity to generate new naïve T-cells [46, 47]. As a result, oligoclonal T-cell populations with restricted epitope specificity accumulate and reduce the efficacy of immunosurveillance [48, 49]. In this setting, EBV-driven clonal proliferation of lymphocytes and subsequent transformation to a malignant phenotype may be facilitated. Certain immunosuppressants may additionally promote EBV-directed lymphocyte proliferation. For example, methotrexate directly activates EBV early promoters resulting in activation of EBV replication [50] while cyclosporine-A induces oxidative stress that in turn directly activates EBV [51]. As a result, lymphoproliferation may be preferentially driven at sites where latently infected EBV lymphocytes are more prevalent.

**Table 2:** Summary of Reported Cases of EBVMCU disease

Age	Sex	Ulcer location	Predisposing factor	Treatment	Response (durability)	Ref
*Iatrogenic immunosuppression-associated EBVMCU*						
53	F	Colon, rectum	Methotrexate + infliximab (CD)	Reduced IS	PD→HL	[75]
56	F	Skin (leg)	Methotrexate (PM)	Reduced IS	CR (33 m)	[76]
59	F	Eyelid	Methotrexate (RA)	Reduced IS	CR (37 mo)	[76]
60	F	Lip mucosa	Methotrexate (RA)	Reduced IS	CR (72 mo)	[20]^b^
61	F	Skin (leg)	Methotrexate (RA)	R-CHOP	CR (25 mo)	[76]
62	F	Lip, nose, eyelid	Methotrexate (PM)	Reduced IS	CR (1 mo)	[45]
64	F	Buccal mucosa	Methotrexate (RA)	Reduced IS	Died^a^	[76]
65	M	Palate	Methotrexate (RA)	Reduced IS	CR (19 mo)	[77]
69	F	Colon	Methotrexate (RA)	NR	NR	[20]^b^
76	F	Eyelid	Methotrexate (RA)	Reduced IS	CR (24 mo)	[76]
80	M	Tongue base	Methotrexate (RA)	NR	NR	[20]^b^
80	F	Skin (arm)	Methotrexate (RA)	Reduced IS	CR (60 mo)	[20]^b^
81	F	Tongue	Methotrexate (RA)	Reduced IS	CR (12 mo)	[78]
42	M	Oral mucosa	Azathioprine (sarcoidosis + MG)	NR	NR	[20]^b^
63	M	Skin (perianal)	Azathioprine (CD)	Reduced IS	CR (6 weeks)	[79]
75	F	Esophagus	Azathioprine (RA)	Reduced IS	CR (17 mo)	[20]^b^
76	F	Buccal mucosa	Azathioprine (Pemphigoid)	Reduced IS	CR (13 mo)	[80]
81	F	Colon	Azathioprine (ITP)	None	PD on diagnosis	[81]
48	F	Tongue	Cyclosporine-A (SLE)	Reduced IS	CR (24 mo)	[20]^b^
64	F	Colon	Cyclosporine-A (HSCT)	Reduced IS	CR (6 mo)	[20]^b^
70	M	Lip	Cyclosporine-A, prednisone (renal transplant)	Reduced IS, surgical excision	CR (111 mo)	[82]
80	F	Rectum	Cyclosporine-A (UC)	Reduced IS	CR (23 mo)	[20]^b^
63	F	Gingiva	Cyclosporine-A, prednisone, MMF (renal transplant)	Reduced IS	CR (8 mo)	[82]
44	M	Tongue	MMF, pred (renal transplant)	Reduced IS	CR (15 mo)	[82]
45	M	Gingiva	MMF (SLE)	NR	NR	[83]
61	M	Esophagus	MMF, pred (renal transplant)	Reduced IS	CR (16 mo)	[82]
70	M	Rectum	MMF, pred (renal transplant)	Reduced IS, rituximab (2 doses) velcade	CR (17 mo)	[82]
18	M	Tonsil, buccal mucosa	MMF, prednisone, tacrolimus (cardiac transplant)	Reduced IS, rituximab (2 doses)	CR (14 mo)	[82]
32	M	Terminal ileum	MMF, prednisone, tacrolimus (bilateral lung transplant)	Reduced IS, rituximab (4 doses)	CR (60 mo)	[82]
*Primary Immunodeficiency-Associated EBVMCU*						
45	F	Gingiva	T-cell deficiency NOS	rituximab (8 doses)	CR (24 mo)	[62]
61	F	Esophagus (multifocal)	hypogammaglobulinemia	rituximab (4 doses), IVIG (monthly), brentuximab (3 cycles)	PD	[63]
*EBVMCU of Unclear Etiology*						
49	F	Gingiva, palate	etiology not established	rituximab (4 doses × 2) RT	PD CR (6 mo)	This case
*EBVMCU secondary to age-associated immunosenescence*						
63	F	Tonsil	Age	NR	NR	[20]
64	F	Tongue, oral mucosa	Age	RT	CR (15 mo)	[20]^b^
68	F	Tongue	Age	None	SR (36 mo)	[20]^b^
68	F	Tonsil	Age	R-CHOP, RT	CR (24 mo)	[20]^b^
73	M	Tonsil, tongue	Age	None	RR (12 mo)	[20]^b^
74	M	Skin (neck)	Age	R-CHOP	CR (24 mo)	[84]
75	F	Skin (arm)	Age	NR	NR	[20]^b^
76	M	Tongue base	Age	None	SR (12 mo)	[20]^b^
79	M	Skin (cheek)	Age	None	SR (25 mo)	[20]^b^
80	F	Palate	Age	RT	CR (60 mo)	[20]^b^
81	F	Palate	Age	None	SR (14 mo)	[85]
82	F	Lip	Age	None	RR (NR)	[20]^b^
82	M	Lip	Age	None	SR (NR)	[20]^b^
84	F	Tongue, floor of mouth	Age	None	SD (5 mo)	[20]^b^
85	F	Tonsil	Age	RT	CR (3 mo)	[20]^b^
88	F	Tongue base	Age	None	RR (24 mo)	[20]^b^
88	M	Skin (chest)	Age	None	SR (3 mo)	[20]^b^
89	M	Tongue base	Age	NR	NR	[20]^b^
89	M	Lip, skin (scalp)	Age	resection	NR	[86]
101	M	Tonsil	Age	R-CHOP	CR (12 mo)	[20]^b^

*F* female, *M* male, *Ref* reference, *CD* Crohn’s disease, *RA* rheumatoid arthritis, *PM* polymyositis, *MG* myasthenia gravis, *ITP* immune thrombocytopenia, *SLE* systemic lupus erythematosus, *HSCT* hematopoietic stem cell transplant, *SOT* solid organ transplant, *UC* ulcerative colitis, *HL* Hodgkin lymphoma, *NOS* not otherwise stated, *Pred* prednisone, *MMF* mycophenolate, *IS* immunosuppression, *R*-*CHOP* rituximab, cyclophosphamide, doxorubicin, vincristine, prednisone, *RT* radiotherapy, *IVIG* intravenous immunoglobulin, *PD* persistent disease, *CR* complete response, *NR* not reported, *SR* spontaneous regression, *RR* relapsing remitting, *Mo* months

^a^Died soon after diagnosis from myelitis and sepsis

^b^Cases are also included in a second published series of EBV-associated lymphoproliferative disorders [87]

### EBVMCU management and unanswered questions

The first case series in which EBVMCU was described suggested that EBVMCU is a relatively benign condition with a self-limited disease course that generally does not require treatment. Our institutional experience and a review of all published cases suggest that for some, EBVMCU can be a progressive and debilitating condition that requires aggressive therapy. As the Word Health Organization (WHO) has not formally recognized EBVMCU as a unique clinical entity, there are no guidelines or consensus opinions to guide treatment. Therefore, we describe a case of progressive EBVMCU that required aggressive therapy and comprehensively review all other published cases to provide a framework on which to base management decisions.

## Case presentation

### Initial presentation

A forty-nine year old homeless woman with a 35 pack year cigarette smoking history and no significant medical problems presented to primary medical care with complaints of painful oral ulcerations on her gums and palate that had been present for at least 6 months. Her review of systems was unremarkable including an absence of constitutional symptoms, infectious symptoms, rheumatologic symptoms, lymphadenopathy, or history of recurrent infections, as well as a lack of history of or risk factors for HIV or immunosuppression. Her physical exam was notable for hypertrophic gums, a 1 cm ulceration adjacent to the right upper incisor, and a palatal ulceration near the left upper molars. She did not have palpable adenopathy, hepatosplenomegaly, rashes, joint tenderness, or joint effusions. Her differential diagnosis at time of presentation included trauma (necrotizing sialometaplasia), infection (herpes simplex virus/HSV, coxsackie virus, human immunodeficiency virus/HIV, syphilis, tuberculosis), autoimmune (systemic lupus erythematosus, Behçet syndrome, reactive arthritis, Crohn disease orofacial granulomatosis variant, Sweet syndrome, granulomatosis with polyangiitis, mucous membrane pemphigoid), carcinoma (squamous cell, malignant salivary gland tumor), or hematologic malignancy (B-cell lymphoma, T-cell leukemia/lymphoma).

### Evaluation

**Fig. 1:**
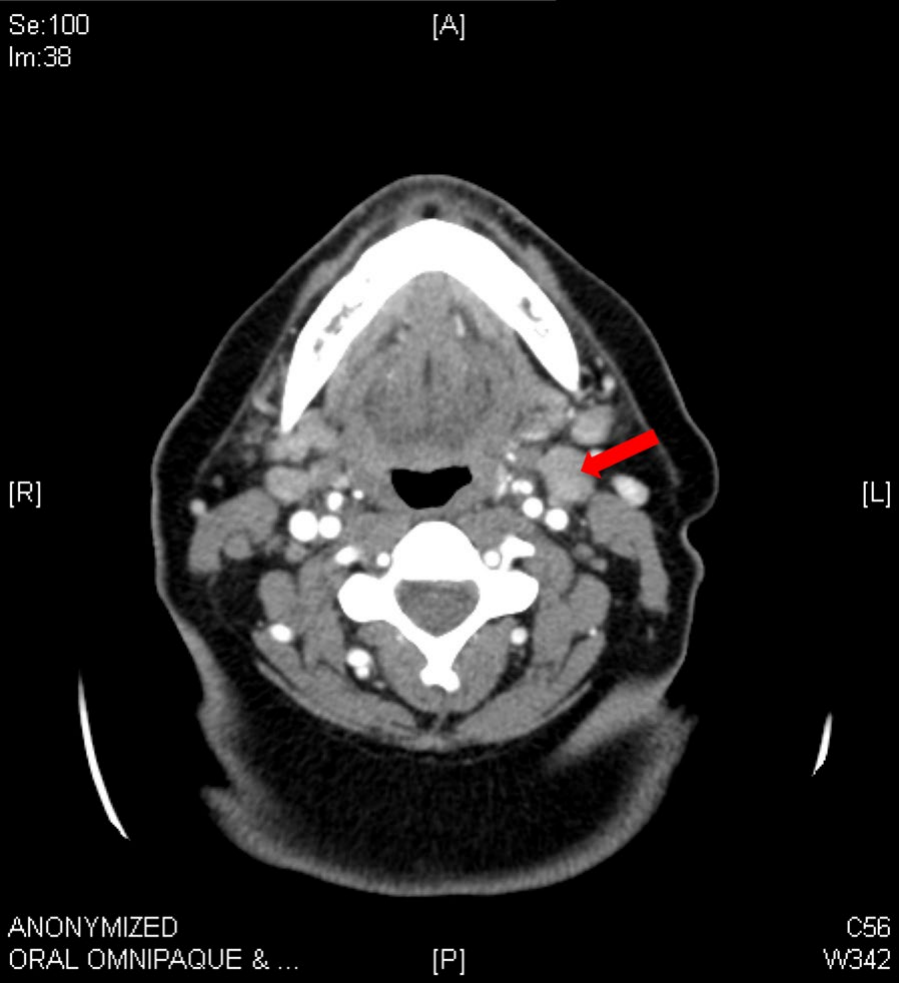
CT of neck demonstrating bilateral diffuse lymphadenopathy with the largest lymph node measuring 2.4 cm (*arrow*)

**Fig. 2:**
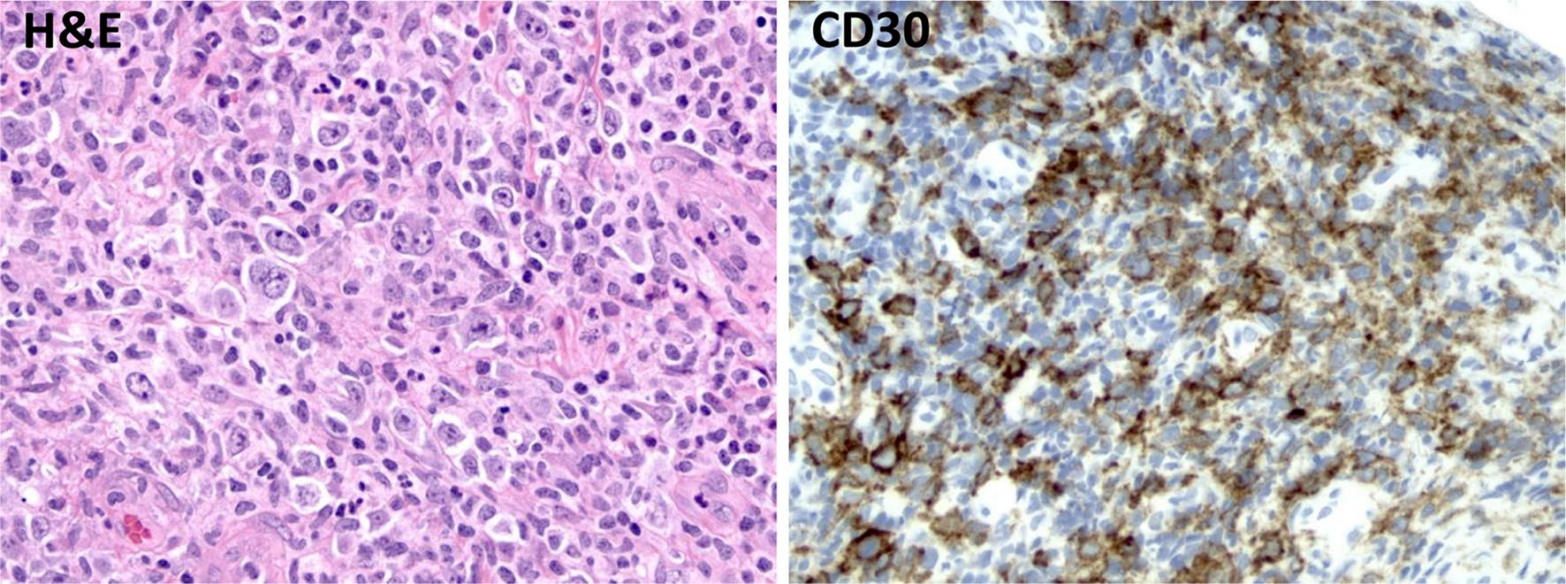
Biopsy of right incisural ulcer and left palatal perimolar ulcer. Hematoxylin and eosin (H&E) stain demonstrating scattered large atypical lymphocytes with occasional Hodgkin Reed-Sternberg (HRS) morphology in a background of mixed inflammatory infiltrate. These large atypical cells are positive for CD30

Diagnostic studies included a normal complete blood count and differential with exception of a mild lymphopenia at 950 cells/μL, normal basic metabolic panel, normal liver function test and lactate dehydrogenase, negative autoimmune screen (antinuclear antibody, anti-neutrophil cytoplasmic antibodies), and negative serologies for HIV and viral hepatitis. Her EBV screen by polymerase chain reaction (PCR) of serum was also negative. Computed tomography (CT) of neck, chest, abdomen, and pelvis demonstrated bilateral diffuse lymphadenopathy in the neck with the largest lymph node measuring 2.4 cm (Fig. 1), but no adenopathy in the hilum, mediastinum, axilla, abdomen, retroperitoneum, pelvis, or inguinal region. The spleen size was normal at 9.2 cm and the abdominal viscera appeared radiographically normal. The patient was referred to oral surgery for biopsy of her right maxillary perimolar lesion and left palatal lesion. Pathologic findings are demonstrated in Fig. 2 and Table 1. Extensive mucosal ulceration was noted with an underlying dense infiltration of small lymphocytes and frequent admixed large atypical lymphocytes including rare Hodgkin and Reed-Sternberg (HRS)-like forms that were positive for CD30 (strong), PAX5 (uniform), CD20 (variable), EBER (uniform), and negative for CD3, CD15, and CD79a. Molecular studies demonstrated a clonal immunoglobulin gene rearrangement. The overall findings were consistent with a clonal EBV-driven, B-cell lymphoproliferative disorder. The diagnostic considerations included EBV-positive mucocutaneous ulcer (EBVMCU) and other EBV-positive B-cell lymphomas including EBV-positive diffuse large B-cell lymphoma, however the patient declined excisional biopsy of cervical lymph nodes and bone marrow biopsy.

### Disease course and management

Given CD20 expression on the large atypical lymphocytes, the patient received four weekly doses of rituximab (375 mg/m^2^) with immediate improvement of symptoms and ulcer regression. Unfortunately this response was short-lived. Within 3 months of completion of rituximab therapy the oral ulcers recrudesced and she declined additional diagnostic studies. Over the next 15 months the oral ulcers increased in extent, with multiple maxillary teeth spontaneously loss, increasing pain, and difficulty opening her mouth resulting in an inability to tolerate solid food, a 36-kilogram unintentional weight loss, and aspiration pneumonia and sepsis.

**Fig. 3:**
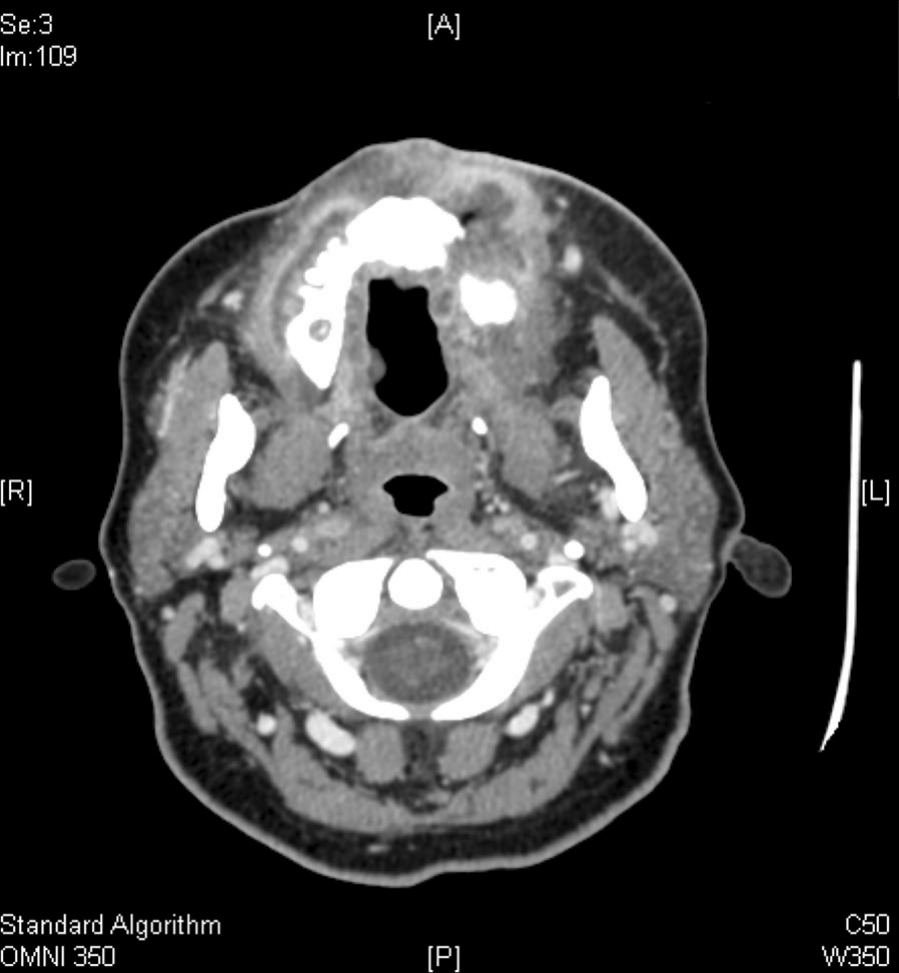
CT of neck demonstrating prominent maxillary gingival and palatal swelling and ulcerations with significant loss of maxillary teeth on the left side

**Fig. 4:**
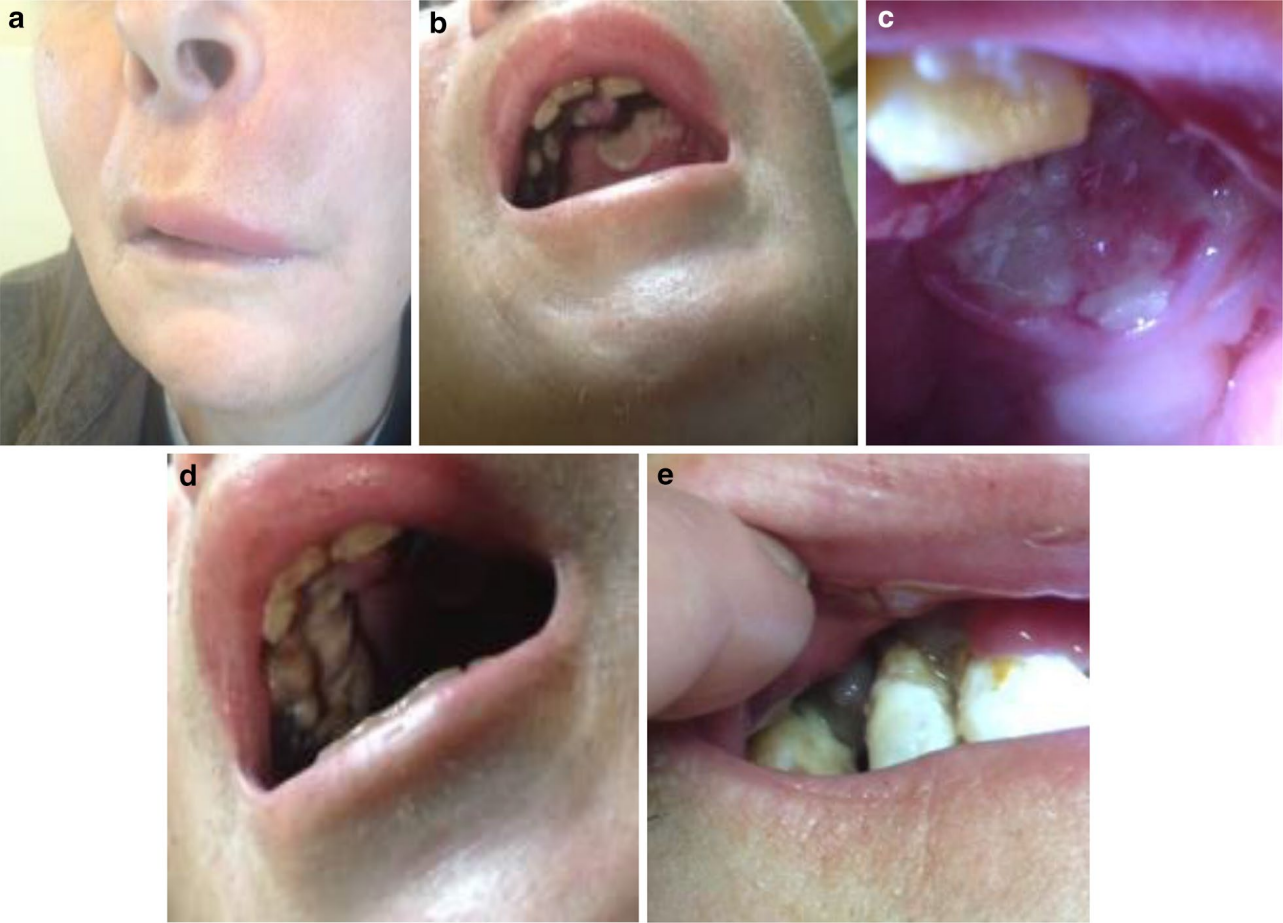
**a** Left sided facial swelling causing lip droop and associated drooling. **b** , **c** Left palatal EBVMCU with associated maxillary teeth loss. **d** EBVMCU along right upper inner gum line extending from incisors to molars. **e** Hypertrophy of right upper gum line

Repeat CT (Fig. 3) demonstrated irregular enhancing soft tissue deep to the left nasolabial region and anterior premaxilla, encroaching on the left nasal vestibule. The tumor invaded the left maxilla. There was diffuse enhancement around the maxilla, palate, and upper oral mucosa. There were shotty bilateral cervical lymph nodes and small subcentimeter bilateral retropharyngeal nodes, but none clearly pathologic. On clinical exam, there was obvious left facial swelling along the left medial cheek and premaxillary region extending into the lip (Fig. 4a). She had limited oral opening due to pain. An ulcerative indurated mass was visible eroding the left maxilla and palate (Fig. 4b, c). It extended to the hard palate-soft palate junction with normal appearing soft palate mucosa. There was fullness and induration suggestive of submucosal extension to the sublabial region and to the buccal mucosa from the lip commisure back to the retromolar trigone. A second ulcer was apparent on the right upper inner gum line extending from the incisors to molars (Fig. 4d) with gum hypertrophy (Fig. 4e) and induration of the right buccal mucosa extending about halfway back. The oral tongue appeared normal. A swab of her ulcers was negative for HSV by PCR and her screen for HIV and EBV was again negative by PCR of peripheral blood. She continued to be resistant to repeat biopsy and bone marrow evaluation. Given the brief response to prior rituximab therapy, four weekly infusions of rituximab dosed at 375 mg/m^2^ were administered; however, the patient failed to experience treatment response. Her oral ulcers persisted and increased in size resulting in an almost total inability to tolerate oral intake with subsequent worsening weight loss, lethargy, and weakness.

**Fig. 5:**
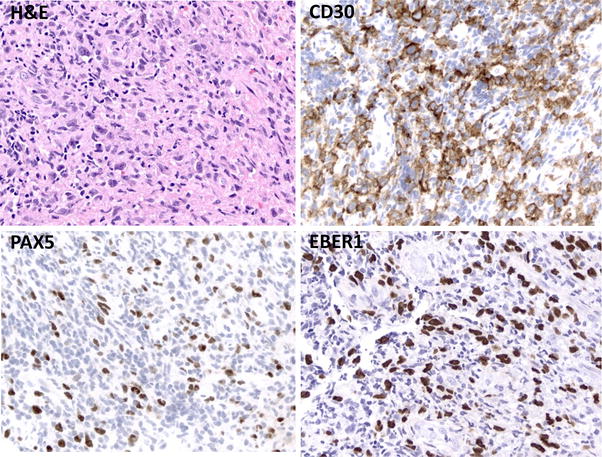
Hematoxylin and eosin (H&E) stain of left palatal ulcer demonstrating a heterogeneous admixture of large atypical lymphocytes with occasional Hodgkin Reed-Sternberg (HRS) morphology, small lymphocytes, and histiocytes. HRS cells express CD30, PAX5, and EBER1 consistent with EBV-infected B-lymphocytes

Her large left palatal ulcer was biopsied with overall pathologic findings similar to that seen in her initial biopsy. Again noted was ulcerated mucosa with underlying dense inflammatory infiltrate of small lymphocytes, eosinophils, neutrophils, histiocytes, and scattered large atypical lymphocytes with rare HRS-like forms. Sheets of large cells were not seen. Large atypical lymphocytes were uniformly positive for CD30, PAX5, MUM1, EBER, EBV LMP (Fig. 5), and negative for CD3, CD10, and CD20 (in the setting of recent rituximab exposure). The Ki-67 proliferation rate of the atypical lymphocytes was 70–80 %. Given the absence of lymphadenopathy or other systemic involvement of disease, a diagnosis of EBVMCU was rendered.

Although HIV testing had been repeatedly negative and the patient had no prior history of recurrent infections or exposure to immunosuppressive agents, a potential underlying immunodeficiency syndrome was considered. Quantitative immunoglobulin (Ig) and T-cell subset analysis was performed and her IgG and IgM were found to be mildly depressed at 544 mg/dL (reference range 620–1490 mg/dL) and 29 mg/dL (reference range 40–350 mg/dL), respectively, while her IgA was normal at 122 mg/dL (reference range 65–420 mg/dL). Her mild hypogammaglobulinemia was likely secondary to recent rituximab exposure, although pre-treatment quantitative immunoglobulins had not been evaluated. Interestingly her CD4 and CD8 T-cell counts were both depressed with an absolute CD4 T-lymphocyte count of 125 cells/μL (reference range 733–2250 cells/μL) and an absolute CD8 T-lymphocyte count of 168 cells/μL (reference range 250–1240 cells/μL). Her CD4/CD8 ratio was relatively preserved at 0.73. Rituximab can cause B-lymphocyte depletion and subsequent hypogammaglobulinemia [52], however it is generally not observed to cause either primary of secondary T-cell depletion [53]. Unfortunately T-cell subset analysis was not performed in our patient prior to rituximab exposure, and we were unable to determine whether she had an underlying immunodeficiency.

**Fig. 6:**
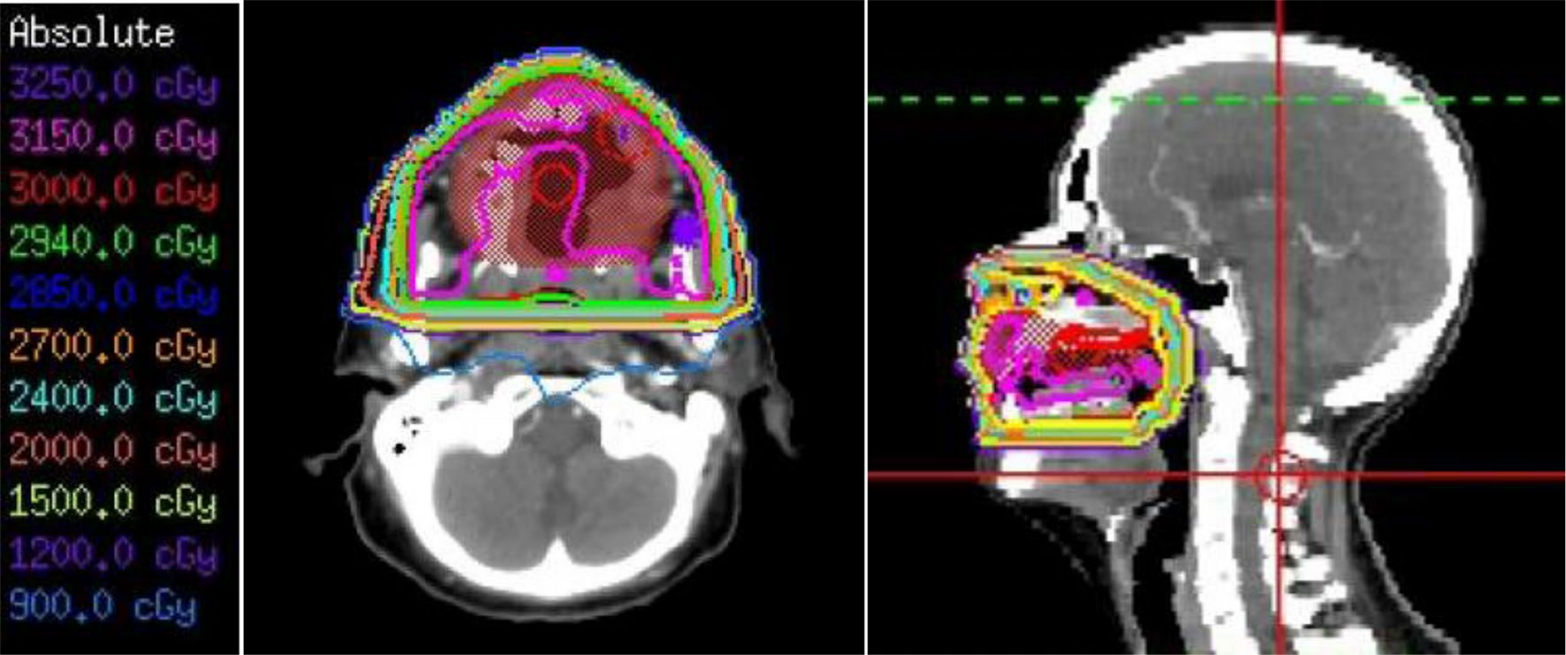
Axial and sagittal views of radiotherapy plan

**Fig. 7:**
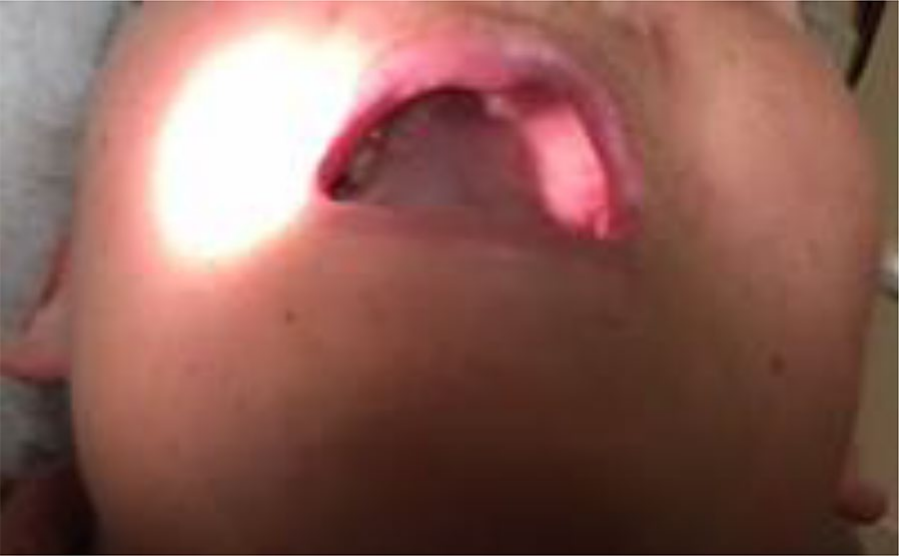
Post-radiation treatment response with resolution of the ulcers along right inner gingiva and left palate. Facial swelling has also resolved

Given our patient’s poor response to recent systemic therapy, radiotherapy was considered as a therapeutic option given case report of excellent clinical response to radiation [20]. Details of radiotherapy including dose were not described; however, extrapolating from radiation approaches with other lymphoproliferative conditions such as low grade lymphomas, we expected doses in the range of 20–45 Gy in conventional fractionation (1.8–2 Gy per fraction) to be associated with a high rate of clinical response and local control with a favorable toxicity profile. Regional nodal dissemination is not characteristic of EBVMCU, so elective nodal irradiation was not deemed necessary. Our patient received external beam radiation therapy using a three-dimensional conformal plan and a three-field approach (opposed lateral and anteroposterior fields; Fig. 6). The gross disease in the oral cavity was treated to 30 Gy in 15 fractions. She experienced a very brisk and complete clinical response with resolution of all oral ulcers and gum hypertrophy (Fig. 7) durable to 6 months.

## Conclusions

### Discussion

EBVMCU is a rare disorder, only recently recognized in 2010 as a unique clinical entity, with 51 total reported cases in addition to this case, and no clear management guidelines to inform care. Although EBVMCU has not been included in the 2008 WHO classification schema of B-cell neoplasms, the presence of discrete ulcer(s) with an underlying polymorphous infiltrate of small lymphocytes, immunoblasts with frequent HRS morphology, plasma cells, eosinophils, and histiocytes is characteristic of the disorder. The immunoblasts are uniformly positive for EBER, CD30, PAX5, OCT-2, and MUM1; have variable expression of CD20, CD45, CD15, CD79a, and BCL-6; and demonstrate monoclonality in a subset. The incidence of EBVMCU is likely under recognized as some lesions may spontaneously regress without pathology being obtained, while other lesions may be misclassified as methotrexate-associated ulcer, Hodgkin lymphoma, or diffuse large B-cell lymphoma, given the presence of large atypical lymphocytes with occasional HRS morphology and immunophenotype.

EBVMCU generally occurs as a solitary lesion but has been reported to be multifocal in 17 % of documented cases including the case we now report. The primary location of EBVMCU is the oropharynx (52 %), with skin (29 %) and GI tract (19 %) occurring less frequently. The recurrent localization of EBVMCU to the oral cavity and GI tract likely reflects the initial site of EBV inoculation in the oropharynx and persistence of latent EBV within lymphocytes of Waldeyer’s ring [44] and gut-associated lymphoid tissue [54]. EBV also has tropism for epithelial cells [55, 56], and 60 % of skin-localized EBVMCU occur on the face at sites overlying the oropharynx. This implicates skin as a sanctuary site for latent EBV and a site of potential EBVMCU formation upon lapse in local immunosurveillance.

EBVMCU has been reported to arise in the setting of iatrogenic immunosuppression (56 %), primary immunodeficiency (4 %), and advanced age (40 %), and is not typically associated with EBV viremia. Local diminution of EBV immunosurveillance as a result of primary or secondary immunosuppression and age-associated immunosenescence is thought to promote EBV-driven lymphoproliferation, clonal lymphocyte expansion, and lymphocyte transformation. Interestingly, in most of the cases of iatrogenic immunosuppression-associated EBVMCU, patients were also of advanced age (median 63 years, mode 80 years, range 18–81 years old) suggesting that age-associated immunosenescence is a potent risk factor for development of EBVMCU. Patients with primary immunodeficiency-associated EBVMCU were of younger age (45–61 years). Although forming generalizations from two patients is problematic, the younger age of onset in these patients may reflect a more profound deficit in immunosurveillance. Interestingly, 62 % of the 52 patients with known EBVMCU have been women, including 62 % of the patients with iatrogenic immunosuppression-associated EBVMCU, 100 % of the patients with primary immunodeficiency EBVMCU, and 55 % of the patients with immunosenescence-associated EBVMCU. The significance of this observation is unclear, as there is not a strong gender bias among other EBV-associated lymphoproliferative disorders with the exception of a male predominance in Burkitt lymphoma and the mixed cellularity subtype of classic Hodgkin lymphoma [57].

EBVMCU has generally been referred to as an indolent, self-limited disease with a benign clinical course that often does not require treatment. Of the patients with iatrogenic immunosuppressant-associated EBVMCU, 68 % experienced complete clinical remission with reduction in immunosuppression alone. Three patients required rituximab in addition to immunosuppression (12 %), one patient underwent surgical excision of their lesion in addition to immunosuppression (4 %), and one patient received aggressive treatment with R-CHOP chemotherapy (rituximab, cyclophosphamide, doxorubicin, vincristine, prednisone), all with complete clinical responses. Two patients died shortly after diagnosis and one patient experienced disease progression despite reduction in immunosuppression, although this individual was receiving dual antimetabolite and anti-TNFα therapy. Taken together, these outcomes suggest that EBVMCU secondary to iatrogenic immunosuppression responds very well to reduction in immunosuppression. Rituximab, a monoclonal antibody against CD20 that results in selective lymphodepletion of B-cells, may provide additional benefit by significantly reducing the local clonal B-cell proliferative response to EBV in EBVMCU. However, given aggressive therapy with R-CHOP was only employed in 1 case, high intensity B-cell directed immunochemotherapy may not be routinely necessary to achieve complete clinical response in EBVMCU secondary to iatrogenic immunosuppression. These treatment strategies are similar to the management approach in PTLD where reduction in immunosuppression [58] with or without rituximab [59], surgical excision of local disease [60], and CHOP [61] have demonstrated overall excellent response rates.

Among the patients with age-associated immunosenescence, 59 % did not receive treatment, of which six experienced spontaneous remissions without intervention (35 %), three experienced a relapsing-remitting disease course (18 %) and one demonstrated prolonged, but stable disease (6 %). Seven patients required treatment (41 %), of whom one underwent surgical resection, three received radiation therapy, two received R-CHOP, and one received R-CHOP + radiation therapy, all with complete clinical responses. Collectively, only 37.5 % of patients in this older population demonstrated a benign clinical course. These outcomes suggest that EBVMCU associated with age-related immunosenescence responds to treatment with radiation therapy or R-CHOP and is associated with sustained disease response. Spontaneous disease remission has been observed in six of 10 untreated cases, however with this small number of patients it is difficult to conclude that age-associated EBVMCU has a high likelihood of spontaneous remission without treatment.

Two cases of EBVMCU occurring in individuals with primary immune deficiency have been published, and in both cases individuals required treatment. One patient had a deficiency of cell-mediated immunity, while the other had a defect in humoral immunity. The patient with a primary T-cell deficiency had severely depressed CD4 T-cell (76 L/μL, reference range 354–1526 L/μL) and CD8 T-cell (71 L/μL, reference range 318–1458 L/μL) counts in the setting of systemic lupus erythematosus that had not required treatment for 10 years [62]. She had quantitatively normal B-cell and natural killer cell counts, and was found to have a mosaic X chromosome abnormality (46,XX,delXq27; 46,XX). Quantitative immunoglobulin testing was not reported. Treatment with 8 doses of rituximab (375 mg/m^2^) resulted in complete clinical remission of her EBVMCU despite persistence of her T-cell deficiency.

The patient with primary hypogammaglobulinemia had normal quantitative lymphocyte counts and peripheral blood lymphocyte phenotyping by flow cytometry, however had markedly reduced IgG (351 mg/dL, reference range 700–1600 mg/dL), IgM (26 mg/dL, reference range 40–230 mg/dL), and IgA (9 mg/dL, reference rage 70–400 mg/dL) levels in the setting of a history of type 1 diabetes mellitus and recurrent infections since young adulthood [63]. She was treated with 4 weekly doses of rituximab (375 mg/m^2^) and monthly intravenous immunoglobulin infusions (600 mg/kg) and experienced symptom improvement. Unfortunately her symptoms returned within 3 weeks of completing rituximab therapy and she experienced disease progression despite the addition of brentuximab vedotin (three doses at 1.8 mg/kg every 3 weeks), an antibody–drug conjugate that delivers a microtubule-directed antineoplastic agent to CD30-positive cells and has been FDA-approved for relapsed Hodgkin lymphoma [64] and has been successfully used to treat PTLD [65]. The authors indicated that they were exploring potential donor-derived EBV-specific T-lymphocyte treatment for this patient.

Both cell-mediated immunity and humoral immunity are required to control EBV-driven lymphoproliferation, and deficiency in cytotoxic CD8 T-cell function, effector CD4 T-cell function, or in immunostimulatory gammaglobulin production enables EBV persistence (reviewed in [66]). It is interesting that in both of these cases of primary immunodeficiency-associated EBVMCU, individuals had an underlying autoimmune disorder. Primary immunodeficiency has an established association with autoimmune disorders [67], including systemic lupus erythematosus [68] and type I diabetes mellitus [69], as well as lymphoproliferative disorders [70]. However, it is unclear whether a causal relationship exists between immunodeficiency and autoimmunity in these two individuals.

We question whether our patient may have an occult T-cell deficiency of unclear etiology given her significantly reduced CD4 T-cell and moderately reduced CD8 T-cell counts. Unfortunately further evaluation of immunodeficiency in our patient is not currently possible as she is homeless, without contact information, and has not returned for medical monitoring or care. Our patient experienced an initial, but short-lived clinical response after rituximab monotherapy. This was followed by significant disease progression over 2 years that was refractory to second rituximab challenge, however responded to 30 Gy fractionated external beam radiation therapy that has been maintained for 6 months, with long-term follow-up currently not available. Importantly, during the nearly 4-year duration of her disease, our patient experienced substantial pain, debilitation, disability, and malnutrition. Although limited to two case reports and potentially our patient, the overall clinical course of primary immunodeficiency-associated EBVMCU disease appears to be more aggressive, multifocal (66 %), with an earlier age of disease onset, and a requirement for directed therapy (rituximab) that may require treatment escalation (brentuximab vedotin, radiation therapy).

Additional treatment strategies that directly target EBV have been explored in other EBV-associated lymphoproliferative disorders. For example, antiviral therapy which targets EBV-specific thymidine kinase has been generally ineffective at controlling EBV-driven B-cell proliferation during viral latency, as it has no effect on EBV episomal DNA and only affects EBV-infected cells in lytic phase in which viral DNA is linearized [71]. Adoptive transfer of EBV-specific cytotoxic T-cells (EBV-CTLs) to reconstitute immunity to EBV has also been investigated. Both autologous and fully or partially human leukocyte antigen-matched allogeneic CTLs have been engineered ex vivo to recognize latent EBV antigens. This strategy has been effective in the treatment of PTLD [72, 73] and classic Hodgkin lymphoma [74], however the cost and time required generating EBV-CTLs is currently a significant limitation to the use and expansion of this technology. It is unclear if these treatment strategies will be effective in cases of progressive and treatment-refractory cases of EBVMCU such as the case reported by Kleinman [63].

All together, these 52 cases suggest that EBVMCU can be a benign and self-limiting condition in some, however at least 29 % require aggressive treatment with CD20- or CD30-directed antibody therapy, local radiation therapy, local surgical excision, systemic chemotherapy, or a combination of these modalities, with an anticipation of durable complete remissions in most. Without aggressive treatment, 16 % of patients reported to date have experienced persistent disease, a relapsing-remitting course, disease progression, or death, while two patients have experienced disease progression despite antibody-directed therapy. Evaluation of all known case reports of EBVMCU including our patient experience suggests that EBVMCU can have a heterogeneous disease presentation, with self-resolving disease in some, but persistent and progressive disease in others requiring aggressive local or systemic therapy. Certainly the morbidity experienced by our patient was sobering.

## Conclusions

In summary, EBVMCU is a rare EBV-associated lymphoproliferative disorder that is likely under reported secondary to its recent recognition and morphologic and immunophenotypic similarities to Hodgkin lymphoma and diffuse large B cell lymphoma. Therefore, we suggest consideration of its inclusion in the next iteration of the WHO classification of lymphoproliferative disorders. This disease is characterized by solitary or multifocal ulcers of the oropharynx, gastrointestinal tract, and skin that result from EBV-driven proliferation of B- and T-lymphocytes in the setting of local lapse in immunosurveillance secondary to iatrogenic immunosuppression, primary immunodeficiency, or age-related immunosenescence. The disease course is heterogeneous, with some patients experiencing spontaneous remissions or complete clinical response to reduction in their immunosuppressive therapies. However for others, EBVMCU can be a persistent, debilitating disease that requires aggressive therapy with CD20- or CD30-directed antibody therapy, local radiation therapy, local surgical excision, systemic chemotherapy, or a combination of these therapies. Disease response to local or systemic therapy is generally excellent (93 %) and sustained (reported durability on follow-up from 3 to 111 months). The care of the patient with EBVMCU is complex. Treatment decisions are not evidence-based and no expert opinion exists. Therefore, we have summarized the limited published experiences with EBVMCU disease to provide a framework from which to guide management.

## Abbreviations

BCL-6: B-cell lymphoma 6
CD: cluster of differentiation
CT: computed tomography
CTL: cytotoxic T-lymphocyte
dL: deciliter
DNA: deoxyribonucleic acid
EBER: Epstein–Barr virus-encoded small ribonucleic acids
EBV: Epstein–Barr virus
EBVMCU: Epstein–Barr virus-positive mucocuatneous ulcer
FDA: Food and Drug Administration
GI: gastrointestinal
Gp: glycoprotein
Gy: gray
HIV: human immunodeficiency virus
HRS: Hodgkin and Reed-Sternberg
HSV: herpes simplex virus
Ig: immunoglobulin
LMP1: latent membrane protein 1
Mg: milligram
MHC: major histocompatibility complex
MUM1: melanoma associated antigen mutated 1
NK: natural killer cell
OCT-2: octamer transcription factor-2
PAX5: paired box protein 5
PCR: polymerase chain reaction
PTLD: post-transplant lymphoproliferative disorder
R-CHOP: rituximab- cyclophosphamide, doxorubicin, oncovin/vincristine, prednisone
RNA: ribonucleic acid
TNFα: tumor necrosis factor alpha
WHO: World Health Organization
